# Chills and Fever

**Published:** 1871-11

**Authors:** 


### CHILLS AND FEVER

Prevail very generally in the fall of the year, over large sections of country. Scattering cases are liable to occur anywhere. These arise from individual indiscretions; but where large numbers of persons in communities are attacked, there some general cause must prevail. This cause has been attributed for ages to "miasm," an emanation from the earth so subtle in its character, that for more than a century the greatest skill of the ablest chemists was not able to detect its nature or define its quality. A bottle of air taken from the most deadly localities was submitted to the most careful and searching analysis without the detection of anything solid, gaseous, or liquid; nothing could be found in the bottle but air, thin air. But the microscope has come to the aid of the alembic, and has discovered in this, the miasmatic air, multitudes of living things. When bottles of this air were taken from the banks of a southern bayou, and placed in the chamber of a man in Chicago by Dr. Salisbury, he was taken with chills and fever in a few days, and these living things were found on his tongue and within his mouth; while not a single one was to be found all over the city, except in that one man's mouth, in his chamber, and in the bottles. Whether this life is animal or vegetable, is a matter of dispute, yet it seems capable of producing chills and fever; but whether animal or vegetable, the laws which regulate the action of miasm on the human system remain the same, and the mode of production, or the causes of the generation of this miasm, remain unchanged, and these laws have been determined and described with wonderful accuracy. This miasm results from warmth, moisture, and vegetation combined; if one is absent, miasm is not formed; vegetable matter will not decay unless there is moisture, it will dry up; it will remain under water a thousand years without decay, as witness the wooden piers of ancient bridges, as sound to-day as when they were driven by Adam's grandson, or somebody else who lived a long time ago. The heat must act on the moisture before miasm becomes a product. This miasm, to be injurious, must be taken into the system by breathing into the lungs, or by swallowing into the stomach. But cold, as the "first frosts," which are everywhere known to make it innocuous, condenses this miasm, makes it so heavy that it falls to the surface of the earth, and can be neither breathed nor swallowed; on the other hand, heat so rarefies the air in which this miasm is contained, that it carries it up towards the clouds, where it is no more breathed than if it laid immediately on the surface of the earth. Hence heat and cold are antagonistic to the disease-producing effects of miasm on the human body. To freeze it out is expensive, but to antagonize it by heat is possible, is everywhere practicable.

From an hour after sundown to an hour before sunrise, the cold causes it to settle on the surface of the earth. An hour after sunrise and until an hour before sunset, as a general rule, it is too high above our heads to injure us, in consequence of the heat of the weather.

As the heat must be over eighty degrees for several days to generate miasm, it follows that the time during which we are required to battle with it is at sunrise and sunset during the spring and fall months. But to make it safe from the first blade of grass in spring until the killing frosts of autumn, dress by a cheerful blazing fire, and take breakfast before going outside of the door; come home before sundown, take your supper before its setting, by the same cheerful blazing hearth, then go and do what you please. You may sleep under a tree, or on a swinging limb, and defy fever and ague for a century, if you only keep warm, abundantly warm.

